# Challenges and Opportunities in One Health: Google Trends Search Data

**DOI:** 10.3390/pathogens12111332

**Published:** 2023-11-09

**Authors:** Lauren Wisnieski, Karen Gruszynski, Vina Faulkner, Barbara Shock

**Affiliations:** 1 Richard A. Gillespie College of Veterinary Medicine, Lincoln Memorial University, Harrogate, TN 37752, USA; karen.gruszynski@lmunet.edu (K.G.); vina.faulkner@lmunet.edu (V.F.); 2 School of Mathematics and Science, Lincoln Memorial University, Harrogate, TN 37752, USA; barbara.shock@lmunet.edu

**Keywords:** google trends, disease prediction, lyme disease, lyme, big data, one health, negative binomial, expanding window, zoonotic disease, tick-borne disease

## Abstract

Google Trends data can be informative for zoonotic disease incidences, including Lyme disease. However, the use of Google Trends for predictive purposes is underutilized. In this study, we demonstrate the potential to use Google Trends for zoonotic disease prediction by predicting monthly state-level Lyme disease case counts in the United States. We requested Lyme disease data for the years 2010–2021. We downloaded Google Trends search data on terms for Lyme disease, symptoms of Lyme disease, and diseases with similar symptoms to Lyme disease. For each search term, we built an expanding window negative binomial model that adjusted for seasonal differences using a lag term. Performance was measured by Root Mean Squared Errors (RMSEs) and the visual associations between observed and predicted case counts. The highest performing model had excellent predictive ability in some states, but performance varied across states. The highest performing models were for Lyme disease search terms, which indicates the high specificity of search terms. We outline challenges of using Google Trends data, including data availability and a mismatch between geographic units. We discuss opportunities for Google Trends data for One Health research, including prediction of additional zoonotic diseases and incorporating environmental and companion animal data. Lastly, we recommend that Google Trends be explored as an option for predicting other zoonotic diseases and incorporate other data streams that may improve predictive performance.

## 1. Introduction

Google Flu Trends (GFT) was a service operated by Google to predict outbreaks of flu and was discontinued in 2015 due to inaccurate predictions. GFT trends overestimated flu prevalence by over 50% in 2011–2012, which some researchers blamed on the increased media coverage and google searches for “swine flu” and “bird flu” [1]. A recent study indicated that a simple heuristic model predicted flu incidence better than the GFT black box algorithm [2]. However, Google Trends may still have potential to be an affordable, timely, robust, and sensitive surveillance system [3] with refinement of search terms, monitoring and updating of the algorithm, and use of additional data streams [1,4]. Google Trends data have been evaluated for their correlation with multiple zoonotic diseases, including Zika [5], salmonellosis [6], encephalitis [7], and Lyme disease [8]. These correlative studies show promise, although the use of Google Trends data for zoonotic disease prediction is underutilized.

Lyme borreliosis (Lyme disease) has been deemed a public health crisis and is reported at epidemic levels in certain geographic areas and is spreading to new geographic areas. Lyme disease is caused by the spirochete bacterium *Borrelia burgdorferi* and is vectored by *Ixodes scapularis* and *I. pacificus* (black-legged ticks) in the eastern and western United States, respectively [9,10]. The wildlife reservoirs for *Borrelia* include rodents in the genera *Peromyscus*, *Sciurus* and *Tamias*. The incidence and prevalence of Lyme disease depends on reservoir and other wildlife populations, environmental factors, tick seasonality and behavior, and landscape level habitat changes as well as other drivers [11,12]. Host response to infection can cause neurologic, cardiovascular, arthritic, and dermatologic issues throughout the stages of infection [13]. Early clinical signs include erythema migrans and early infections are frequently associated with neurological disease as well as arthralgia, fever, and headache [13,14]. Disseminated infection frequently includes arthritis as well as other complications. Most patients recover after treatment with antibiotics. Although approximately 30,000 cases of Lyme disease are reported to the CDC annually, estimated annual diagnosed cases are much higher, i.e., >450,000 [15,16], representing substantial economic and disease burden. Due to the expanding range of *Borrelia* in the United States, diagnosis should be considered based on clinical signs and history of exposure, especially in emerging areas [17].

Here, we demonstrate how Google Trends data can be used as a tool for the prediction of Lyme disease cases. We build on previous work from Kim et al., 2020 [18], who investigated the spatial-temporal associations of monthly Lyme disease incidence and Google Trend search data in the United States from 2011 to 2015 and found that there were similar patterns between the search patterns and incidence at the state-level and at the metro-level in Texas. However, the authors noted that validation of the method is needed due to the non-specific symptoms of Lyme that correspond to other conditions. In addition, the analysis was correlative rather than predictive. Therefore, we aimed to validate their findings by analyzing search terms for diseases with similar symptoms, including fibromyalgia, multiple sclerosis, and arthritis. In addition, we aimed to build predictive models for Lyme disease incidence by state to improve the utility of the models. The results of this paper serve as a case study for using Google Trends search data as a tool for the prediction of zoonotic disease incidences and to highlight benefits and potential barriers to using it as a tool.

## 2. Materials and Methods

### 2.1. Data Retrieval

The Lincoln Memorial University Institutional Review Board approved the study protocol (1075 V.0). Monthly state-level Lyme disease case count data from 2010 to 2021 were requested from multiple state public health departments or obtained from online repositories. Only states with 10 or more cases in 2019 were considered [19]. The final states included in the analysis were based on convenience, lack of missing or concerns regarding protection of individually identifiable health information, and data availability.

Google Trends search data were downloaded using the ‘gtrendsR’ package in R version 4.0.2. [20,21]. Google Trends reports data as “interest over time,” which ranges from 0 to 100 and represents the terms current interest level compared to its highest interest level (at 100). Search terms were selected by evaluating previous research [18] and through discussions of the primary literature and colloquial knowledge by the study team. The final list of search terms included terms for Lyme disease (“Lyme”, “Lyme disease”, and “Lymes”), tick (“seed tick”), symptoms of Lyme disease (“tick bite”, “bone pain”, “stiff neck”, “circular rash”, “brain fog”, tick fever”, “tick rash”, “bulls eye”, “droopy eye”, “muscle ache”, and “lethargy”), and diseases with similar symptoms to Lyme disease (“bells palsy”, “arthritis”, “fibromyalgia”, “multiple sclerosis”, “chronic fatigue”, “Summer Flu”, and “Rocky Mountain Spotted Fever”). The search terms for diseases with similar symptoms were used to test specificity of the search terms for Lyme disease and its symptoms for predicting Lyme disease case count.

### 2.2. Statistical Analysis

Twelve-month expanding window negative binomial regression models were built using the ‘rolling’ command in Stata version 17.0 [22] to predict the number of Lyme disease cases, after determining the data were over-dispersed. Separate models were built by search term, so in total, 22 models were tested. Predictors in the model included the current search term interest and a 12-month lag term to adjust for seasonal differences. Predictive ability was assessed in the test dataset via root mean squared error (RMSE) and through plots of the observed versus predicted counts. RMSE was calculated using the following equation for each observation (*i*), within state (*j*), within year (*k*), and within month (*l*) [23]:

$$\mathrm{RMSE}=\sqrt{\frac{1}{n}\;\overset{n}{\underset{ijkl=1}{\sum }}{\left(O_{i}-\hat{E_{i}\;}\right)}^{2}}$$

where *O* is the observed Lyme disease case count and *E* is the expected, or predicted, case count. RMSE can be interpreted on the same scale as the outcome (Lyme disease case count) and is the average deviation of expected versus observed counts. Therefore, the lower the RMSE, the better the model is at predicting Lyme disease case count.

## 3. Results

**Figure 1:**
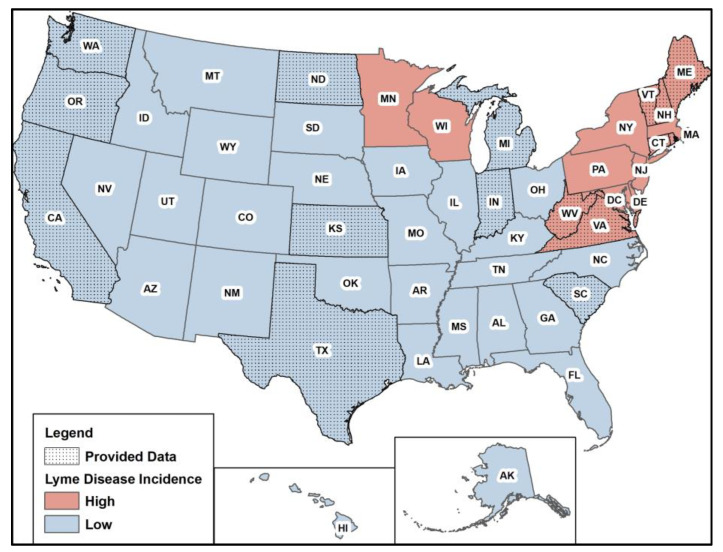
Map displaying states included in analysis (dots) and by high (red) versus low (blue) incidence.

The final sample included data from 16 states (Figure 1). Seven of the sixteen states are considered high-incidence states according to the CDC (https://www.cdc.gov/lyme/datasurveillance/lyme-disease-maps.html, accessed on 2 August 2023). All available data provided from 2010 to 2021 were used for the analysis, and states had variable levels of missing data (Table 1). Data notes and caveats supplied from health departments are listed in Supplementary File S1. Washington had the lowest amount of missing data, and Virginia had the highest amount of missing data. Descriptive statistics of the average monthly Lyme disease case counts stratified by state are summarized in Table 1.

### Predictive Models

The strongest predictive terms were terms for Lyme disease, including “Lyme Disease”, “Lymes”, and “Lyme”, which had the lowest overall RMSE values (Table 2). The RMSE for “Lyme Disease” was 49.8, which can be interpreted as follows: on average, the model with search terms for “Lyme Disease” predicted within 49.8 cases of the actual case count. The interpretation and evaluation of RMSE depends on the scale of the outcome; therefore, RMSEs are expected to be smaller for low-incidence states. For example, for a high-incidence state where the model performed well (New Hampshire, range of 2–527 cases per month), the average RMSE was 73.1, meaning that on average the model predicted within 73.1 cases of the actual case count. For a low-incidence state where the model performed well (North Dakota, range of 0 to 21 cases a month), the RMSE was 3.8, meaning that on average, the model predicted within 3.8 cases of the actual case count. The worst performing terms were “tick bite”, “tick rash”, and “Rocky Mountain Spotted Fever”, indicating that Google Trends terms have some specificity for predicting Lyme disease. However, the average RMSEs ranged from 49.8 to 83.4 for all search terms, indicating that the search terms for Lyme disease were more predictive for Lyme disease, but not by a large margin.

**Figure 2:**
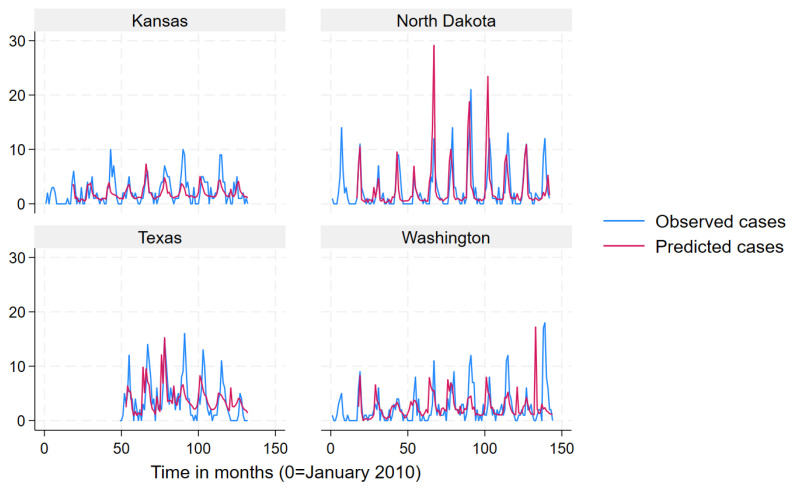
Observed (blue line) versus predicted (red line) monthly Lyme disease case counts using the search term “Lyme disease” for very low-incidence states.

**Figure 3:**
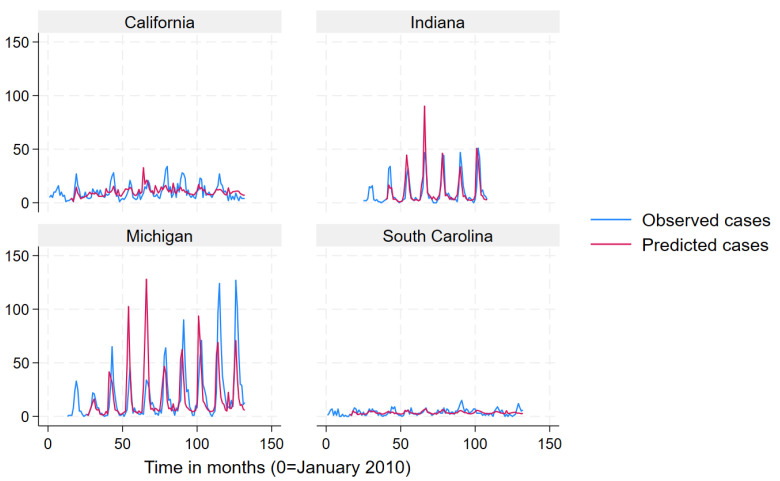
Observed (blue line) versus predicted (red line) monthly Lyme disease case counts using the search term “Lyme disease” for low-incidence states.

**Figure 4:**
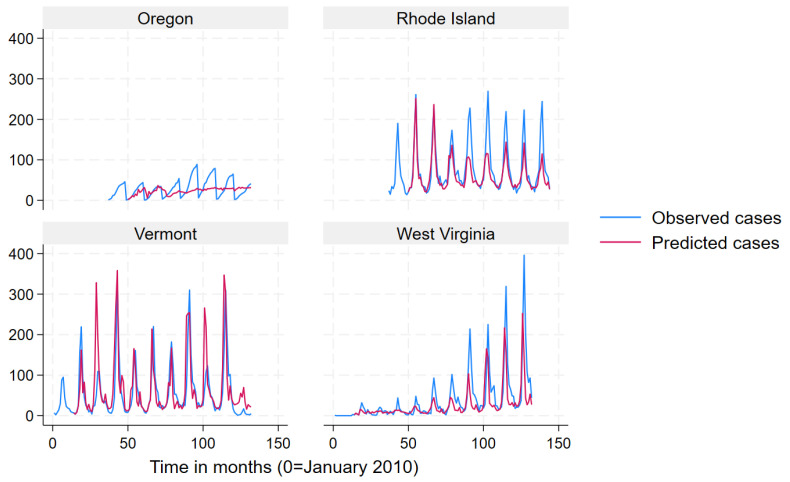
Observed (blue line) versus predicted (red line) monthly Lyme disease case counts using the search term “Lyme disease” for high-incidence states.

**Figure 5:**
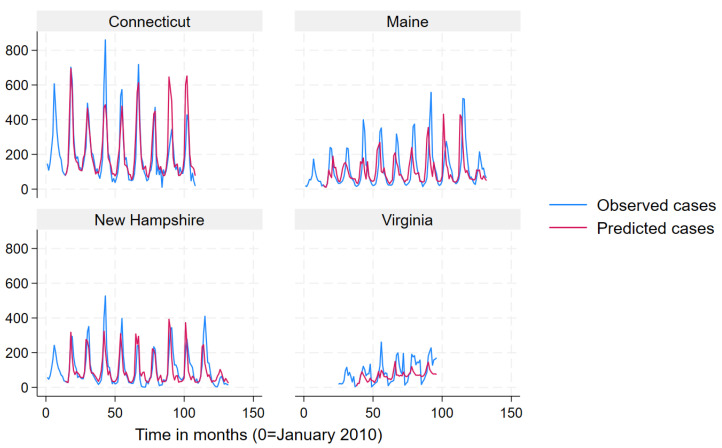
Observed (blue line) versus predicted (red line) monthly Lyme disease case counts using the search term “Lyme disease” for very high-incidence states.

We used a mean monthly Lyme disease case count as calculated from the data to define states into “very high incidence” (>78.6), “high incidence” (19.3–78.6) “low incidence” (3.9–19.2) and “very low incidence” (<3.9) categories for data presentation in Figure 2, Figure 3, Figure 4 and Figure 5. Prediction intervals are presented in Supplementary Table S1. Results for the best performing term “Lyme Disease” are presented. In some states and across all incidence levels, the predicted case counts closely follow the observed case counts, which indicated good predictive ability. The model appeared to perform best for North Dakota, Indiana, Michigan, Vermont, Connecticut, Maine, and New Hampshire. However, the model appeared to perform poorly for Kansas, Texas, Washington, California, Oregon, Rhode Island, and Virginia.

## 4. Discussion

Google Trends data are freely available and downloadable, which provides accessibility for researchers, epidemiologists, and health departments. Google Trends was used by the CDC for the prediction of yearly influenza cases, but eventually they discontinued use due to low predictive ability [1]. In this study, we assessed the predictive ability of Google search terms for monthly Lyme disease case count at the state level. We found that the models produced accurate predictions for many states, as demonstrated by the closeness of the predicted and observed case counts. In addition, the most predictive terms for Lyme disease case count were terms for Lyme disease, which indicates specificity for Google trends search term in predicting Lyme disease case counts. However, Google Trends underperformed in multiple states and there were no clear trends across incidence levels. We conclude that Google Trends data have the potential to be used as a tool for zoonotic disease incidence prediction in addition to other surveillance tools.

In this case study, we encountered numerous barriers to using Google Trends data to predict Lyme disease case count. First, Google Trends data may have underperformed in multiple states due to differences in how each health department tracks and reports Lyme disease case data. In the United States, each state health department tracks and reports Lyme disease data and there is not a centralized data system. The health departments then report yearly data to the CDC. Case definitions are not consistent across state or even across time, which may have also affected the performance of the models. Some state departments censor small cell sizes, so we were unable to include those states in the models. A barrier for data acquisition is that the system for requesting data in each state varies. Some states have data readily available for use on their official websites, whereas others require full Institutional Review Board review. Another challenge of using Google Trends data for disease prediction is the geographical units of the Google Trends search data. Google Trends data does not report at the county level, likely due to search volume and data privacy issues. The smallest geographical unit reported is at the metro-level, which is a geographical area that corresponds to a metropolitan area. Unfortunately, this does not correspond directly to county-level data, which is how most health departments report case data. Another challenge is selecting search terms. In the future, we recommend considering regional differences in terminology when selecting Google Trends search terms to potentially improve model performance in states with poor predictions, while also considering search volume. In less-populated states, some of our selected Google Trends search terms did not reach an adequate search volume to use in the models.

A nationwide, centralized data reporting system with standardized definitions for monthly Lyme disease cases would improve the feasibility of utilizing Google Trends for Lyme disease prediction. Currently, the CDC maintains a Lyme disease data dashboard, although the units reported are at the yearly level, which makes finer prediction not possible. Lyme disease cases are now reported at epidemic levels in some areas and there should be urgency in improving access to data [24]. Nonetheless, states could utilize Google Trends search data as a tool in their toolbox for disease surveillance in conjunction with other surveillance techniques, such as physician-reported cases, tick dragging, insurance claims, and wildlife or pet reports. This likely would require collaboration across state departments.

Future studies can determine if we can predict the spread of Lyme disease in new geographical locations and on a finer scale. In addition, future studies should investigate the inclusion of environmental, tick, and companion animal data for model refinement and to consider the full One Health triad. Future studies can also validate the findings of this case study in other zoonotic diseases and determine if the Lyme models could improve over time with additional data. There is a risk that with more media attention on Lyme disease, the models will be less predictive. However, using the expanding window approach where the model retrains every 12 months will help prevent media attention from affecting model performance.

## 5. Conclusions

In this study, we demonstrate the potential use of Google Trends search data for the prediction of monthly Lyme disease case counts at the state-level. Model performance varied by state. We outline challenges for Google Trends disease prediction, such as different case definitions and reporting procedures by state, data availability, and mismatch of Google Trends geographical units with county case counts. However, there are many opportunities for utilizing Google Trends data, as it is a free, publicly available resource and has not yet been tested for predictive ability for many zoonotic diseases. Integration of environmental, tick, and companion animal data is the next step to make it a true One Health model. Incorporating additional data sources may improve predictions for states where Lyme disease search terms did not accurately predict case counts.

## Figures and Tables

**Table 1:** Descriptive statistics for monthly Lyme disease case count by state included in analysis (*N* = 1879 observations).

State	N	Mean	SD	Minimum	Median	Maximum
California	132	10.0	7.0	1	8	34
Connecticut	108	206.0	170.3	11	152.5	860
Indiana	84	10.2	13.1	0	4	51
Kansas	132	2.2	2.3	0	2	10
Maine	132	113.3	112.1	12	71	557
Michigan	120	19.2	25.2	0	9	127
New Hampshire	132	106.1	103.6	2	64	527
North Dakota	140	2.8	3.9	0	1	21
Oregon	96	29.1	22.2	1	25.5	89
Rhode Island	108	78.6	61.9	14	56.5	269
South Carolina	132	3.8	2.8	0	3	15
Texas	84	3.9	3.9	0	3	16
Vermont	131	55.7	64.8	1	28	312
Virginia	72	87.6	63.8	3	78	261
Washington	144	2.5	3.2	0	1	18
West Virginia	132	38.6	60.5	0	17	396

**Table 2:** Root mean squared error (RMSE) of predictions from model predicting monthly Lyme disease case count stratified by Google search term ^1^.

	CA	CT	IN	KS	ME	MI	NH	ND	OR	RI	SC	TX	VA	VT	WA	WV	All
Symptoms																	
Bulls eye	6.8	136.6	13.3	2.3	108.2	25.1	106.2	-	21.9	-	2.7	3.3	63.3	-	3.3	-	63.4
Droopy eye	6.8	168.0	12.8	2.4	114.3	25.2	105.7	-	21.2	-	2.7	3.5	63.4	-	3.3		69.9
Stiff neck	6.9	152.2	13.2	2.3	115.3	24.8	101.2	3.6	21.6	59.1	2.7	3.7	57.2	67.5	3.3	52.9	62.5
Tick bite	6.2	249.2	38.9	2.0	123.9	36.6	130.9	9.1	22.1	45.3	2.5	3.6	61.6	77.2	4.9	58.0	83.4
Tick fever	6.5	159.2	17.8	2.1	101.5	22.3	101.5	-	22.1	61.5	2.7	2.6	58.8	65.7	3.3	61.8	64.9
Tick rash	6.3	197.3	13.0	2.0	105.5	75.2	105.1	-	22.1	62.8	2.7	3.3	62.1	68.7	2.9	46.6	73.7
Similar diseases																	
Arthritis	7.0	161.9	12.3	2.3	110.8	24.1	103.1	3.7	20.8	57.5	2.7	3.8	62.5	70.1	3.3	55.6	64.2
RMSF	6.1	172.6	140.4	1.9	107.0	24.8	101.4	3.6	22.1	53.8	2.7	3.0	58.7	60.5	3.2	57.7	70.1
Summer flu	5.9	167.8	9.6	2.3	114.3	19.9	105.7	3.9	22.4	61.5	2.7	3.0	59.8	65.7	2.6	61.8	65.6
Lyme disease																	
Lyme	6.2	156.4	9.6	2.0	114.4	24.4	69.9	4.9	22.2	32.1	2.6	3.7	60.6	53.5	3.3	42.1	57.3
Lyme disease	6.5	96.8	9.7	2.0	113.5	24.4	73.1	3.8	22.3	38.8	2.6	3.2	61.1	54.4	3.5	44.0	49.8
Lymes	6.4	120.9	8.0	2.1	103.5	23.0	95.3	3.8	23.0	41.5	2.7	3.2	60.9	58.4	3.3	43.5	54.1
Other																	
Seed tick	7.0	168.0	12.6	2.4	114.3	23.3	103.3	-	22.6	61.5	2.8	3.4	55.2	65.7	3.3	61.8	67.9

^1^ Missing values in the table are due to low search volume. RMSF: Rocky Mountain Spotted Fever.

## Data Availability

Restrictions apply to the availability of Lyme disease case data. Supplementary File S1 includes links to data repositories for Connecticut, Indiana, Oregon, and Virginia. Data from the remaining states can be requested from individual health departments. Google Trends search volume data available on request from the corresponding author.

## Supplementary Materials

The following supporting information can be downloaded at: https://www.mdpi.com/article/10.3390/pathogens12111332/s1, File S1: Data notes and caveats; Table S1: Prediction intervals for Lyme disease case counts.
